# An Integrated Detection Based on a Multi-Parameter Plasmonic Optical Fiber Sensor

**DOI:** 10.3390/s21030803

**Published:** 2021-01-26

**Authors:** Gongli Xiao, Zetao Ou, Hongyan Yang, Yanping Xu, Jianyun Chen, Haiou Li, Qi Li, Lizhen Zeng, Yanron Den, Jianqing Li

**Affiliations:** 1 Guangxi Key Laboratory of Precision Navigation Technology and Application, Guilin University of Electronic Technology, Guilin 541004, China; xiaogl.hy@guet.edu.cn (G.X.); 19022202023@mails.guet.edu.cn (Z.O.); 19022304015@mails.guet.edu.cn (Y.X.); 19022202002@mails.guet.edu.cn (J.C.); lihaiou@guet.edu.cn (H.L.); lqmoon@guet.edu.cn (Q.L.); dyr0211@guet.edu.cn (Y.D.); 2 Guangxi Key Laboratory of Automatic Detecting Technology and Instruments, Guilin University of Electronic Technology, Guilin 541004, China; 3 School of Electronic Engineering and Automation, Guilin University of Electronic Technology, Guilin 541004, China; 4 Graduate School, Guilin University of Electronic Technology, Guilin 541004, China; zlzh@guet.edu.cn; 5 Guangdong-Hong Kong-Macao Joint Laboratory for Intelligent Micro-Nano Optoelectronic Technology, Foshan University, Foshan 528225, China; jqli@must.edu.mo

**Keywords:** multi-parameter, graphene, D-type photonic crystal fiber (PCF), biosensor

## Abstract

In this paper, a multi-parameter integrated detection photonic crystal fiber (PCF) sensor based on surface plasmon resonance (SPR) is proposed for its application in detecting temperature, magnetic field, and refractive index. The air holes on both sides of the fiber core were coated with gold film and introduced to the temperature-sensitive medium (PDMS) and magnetic fluid (MF), detecting temperature and magnetic field, respectively. The graphene layer is also presented on the gold film of the D-type side polished surface to improve the sensor sensitivity. The sensor’s critical parameters’ influence on its performance is investigated using a mode solver based on the finite element method (FEM). Simulation results show when the samples refractive index (RI) detection is a range of 1.36~1.43, magnetic field detection is a range of 20~550 Oe, and the temperature detection is a range of 5~55 °C; the maximum sensor’s sensitivity obtains 76,000 nm/RIU, magnetic field intensity sensitivity produces 164.06 pm/Oe, and temperature sensitivity obtains −5001.31 pm/°C.

## 1. Introduction

Compared with traditional optical fiber, photonic crystal fiber (PCF) has attracted more and more researchers’ attention due to its high birefringence, low loss, high nonlinearity, and flexible structure. Photonic crystal fiber (PCF) introduces a stomatal filling structure into the cladding. It fills the pores with different sensitive materials to show photonic crystal fiber sensors’ other sensing characteristics in different environments [1,2,3]. Surface plasmon resonance (SPR) is a physical phenomenon occurring on dielectric and metal surfaces [4,5,6].When the evanescent wave generated by the total internal reflection of incident light appears at the interface of the metal medium and the surface plasmonic wave meeting the phase-matching condition, resonance will occur, resulting in a large amount of incident light energy being coupled to the surface plasmon wave, the incident light energy will sharply decrease, and show a loss resonance peak in the loss spectrum. Because SPR can effectively detect small changes in the refractive index of the surrounding environment, in 1968, Otto combined a prism with a metal film to design the first SPR sensor structure [7], which attracted much attention because of its high sensitivity. However, the design of the Otto structure increases the complexity of the overall system of the sensor. Therefore, in 1971, Kretschmann improved the Otto structure [8] and significantly reduced the structural complexity. However, the prism-based SPR sensor needs complex optical and mechanical components, which leads to its large volume and is not suitable for remote sensing [9]. In 2006, Hassani et al. first combined SPR sensing with PCF and designed a surface plasmon resonance sensor based on photonic crystal fiber [10]. This sensor has a flexible structure, easy phase matching, good single-mode characteristics, and high sensitivity. However, in this structure, both the liquid channel and the metal film are placed in the cladding air hole of the photonic crystal fiber, which requires a high process and is difficult to make. Therefore, in 2012, Ming Tian et al. proposed a model of outer cladding coating based on D-type PCF [11], which placed the liquid and metal film outside the cladding, thus significantly reducing the process difficulty and practical operation complexity. Still, this structure is coated with metallic silver on the side polishing plane, and silver is easy to oxidize, resulting in reduced sensor accuracy. Graphene coatings have a broad prospect in sensor application because of its high surface volume ratio, high electron mobility, and strong absorption. In 2014, Dash J. N. proposed a PCF–SPR sensor coated with graphene on silver film [12]. When graphene is coated on the surface of the silver film, it can not only prevent the silver film from being oxidized, but also improve the sensitivity of the sensor [13].

However, the sensors mentioned above can only detect a single parameter, and if multiple parameters can be seen in one sensor, the integration of the sensor can be effectively improved. In 2015, Shiwei Hua et al. proposed a refractive index and temperature sensor based on D-shaped photonic crystal fiber [14]. Temperature-sensitive liquid toluene is filled in an air hole in the cladding, and the directional coupling effect measures the temperature. In 2017, Hai Liu et al. proposed a PCF temperature and magnetic field two-parameter optical sensor based on directional coupling resonance [15]. In 2019, Ying Yu et al. proposed a two-parameter sensor based on D-type PCF and magnetic field and temperature sensor [16]. The magnetic fluid is deposited on the side polishing plane and filled with ethanol in the air hole. Under the magnetic field of 30~270 Oe and the temperature of 5~65 °C, the sensor can show the magnetic field sensitivity of 0.21 nm/Oe and the temperature sensitivity of −1.25 nm/°C.

Based on the above research background, we propose an SPR D-type PCF sensor integrated with temperature, magnetic field intensity, and refractive index multi-parameter detection in this paper. When graphene is coated on the gold film of the PCF side polishing plane, it can prevent protein denaturation during the detection of biomolecules and improve RI sensitivity. Then, we introduce magnetic fluid and temperature-sensitive medium into the air holes at both ends of the fiber core and use the magneto-optical effect, temperature-sensitive effect, and SPR effect to form a magnetic field sensing channel and temperature sensing channel, to design and realize the multi-parameter integrated detection of temperature, magnetic field strength, and refractive index of D-type PCF SPR sensors. By optimizing the structure, when the distance between air holes is *Λ* = 16 µm, the depth of side polishing is *d_h_* = 0.7 Λ, the diameter of air hole in cladding is *d* = 0.5 Λ, the diameter of channel 1 (magnetic field sensing channel) is *d*_*ch*1_ = 7 µm, the diameter of channel 2 (temperature sensing channel) is *d*_*ch*2_ = 6 µm, the number of graphene layers is *N* = 3, the thickness of metal film on side parabolic surface is *t* = 55 nm, the thickness of the metal film in channel 1 is *t*_1_ = 40 nm, the thickness of the metal film in channel 2 is *t*_2_ = 45 nm, the average sensitivity of the sensor is 17,571 nm/RIU. The maximum sensitivity can reach 76,000 nm/RIU. The structure designed in this paper overcomes the limitation of single-parameter measurements of traditional sensors and realizes the integrated detection of multi-parameters (magnetic field intensity, temperature, and refractive index). The structure design and simulation results can open up a new field for realizing biomedical and multi-function sensor detection.

## 2. Sensor Principle

**Figure 1:**
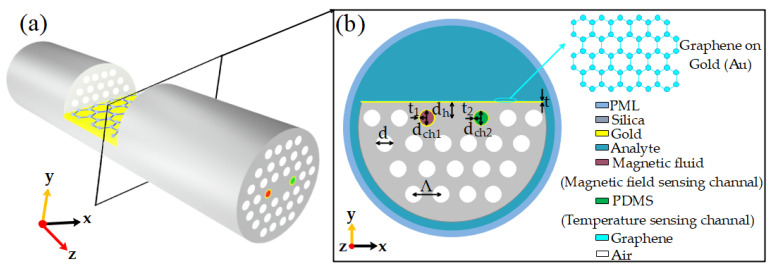
Schematic diagram of a plasmonic optical fiber sensor: (**a**) its three-dimensional structure; (**b**) its two-dimensional cross-section structure.

This paper analyzes the modal characteristics of an SPR D-type PCF sensor with multi-parameter integrated detection of temperature, magnetic field intensity, and refractive index by finite element method. The multi-parameter sensor proposed in this paper is shown in Figure 1. Firstly, the gold film was coated on the PCF cladding polishing plane to form the SPR sensing channel. When the refractive index of the measured liquid changed, the wavelength shift of the SPR loss peak was analyzed to realize the sensor’s refractive index sensing measurement. Then, the sensitivity of the sensor was improved by introducing a graphene layer on the gold film. The air hole on the left side of the fiber core was filled with magnetic fluid Fe_2_O_3_ to form channel 1, and that on the right side of the center was filled with the temperature-sensitive medium PDMS to form channel 2. Channel 1 and channel 2 were symmetrical, and their inner walls were coated with a gold film. They used the magnetic fluid and the temperature-sensitive medium to respond differently to magnetic field changes and temperature changes. The peak values of SPR loss and the wavelength shift of the two channels’ resonance wavelength were different. Then, through the sensitivity matrix method, the simultaneous sensing measurement of temperature and a magnetic field was realized; Finally, a D-type photonic crystal fiber SPR sensor was designed and acknowledged, which integrated multi-parameter detection of temperature, magnetic field, and refractive index.

As shown in Figure 1, the air holes in the optical fiber are composed of 19 air holes arranged in parallel in four layers, in which the air holes in the first layer are arranged in a horizontal straight line with the fiber core and two-channel, and two air holes are distributed on the left and right; there are six air holes in the second layer, five in the third layer, and four in the fourth layer. The outermost air hole center of these four layers of air holes is connected to form half of a regular hexagon. By introducing air holes into the fiber cladding to create a solid photonic crystal fiber, the light wave can be better constrained in the core to gather high field strength in the center of the fiber and finally enhance the nonlinear effect of the fiber.

In the PCF sensor designed in this paper, the substrate material of optical fiber was molten SiO_2_, and the relationship between refractive index (RI) *n* and wavelength *λ* can be defined according to the Sellmeier Equation [17]. The expression is as follows:

(1) $$n^{2}\left(\lambda \right)=1+\frac{0.6961663\lambda^{2}}{\lambda^{2}-{\left(0.0684043\right)}^{2}}+\frac{0.4079426\lambda^{2}}{\lambda^{2}-{\left(0.1162414\right)}^{2}}+\frac{0.8974794\lambda^{2}}{\lambda^{2}-{\left(9.896161\right)}^{2}}$$

The relative dielectric constant of gold film Au is defined by the Drude model [18]. For the graphene layer, the refractive index expression [19] is as follows:

(2) $$n=3+\frac{iC_{1}\lambda }{3}$$

Generally speaking, the thickness of the monolayer graphene is 0.34 nm. For Equation (2), *C*_1_ is constant. Its value is 5.446 µm^−1^, and *λ* is the vacuum wavelength. In this design, the total thickness of graphene was *D* = 0.34 × *N*, where *N* is the number of graphene layers.

Magnetic fluid (MF) is a kind of colloid solution. The changes of external temperature and magnetic field intensity will affect the refractive index of MF [20,21,22]. The relationship between them can be described by the Langevin Function [23]. The magnetic fluid material selected in this paper was water-based Fe_3_O_4_. When the volume ratio of Fe_3_O_4_ to water is 3%, the refractive index of MF is 1.3592, and the thermo-optic coefficient and magneto- optic coefficient are −2.4 × 10^−4^/°C and 4.98 × 10^−5^/Oe, respectively. The function [24] of the magnetic fluid designed in this paper can be described as follows:

(3) $$n_{MF}=1.3592-2.4\times 10^{-4}\Delta T+4.98\times 10^{-5}\Delta H$$

PDMS (Polydimethylsiloxane) is a new type of polymer material. In existing temperature sensors, ethanol is mostly used as a sensitive material to detect the temperature. Compared with the PDMS used in this paper, ethanol is toxic, easy to leak and volatile, and PDMS has good mechanical properties, easy processing and higher thermo-optic coefficient.When the temperature is 20 °C, the thermo-optic coefficient of PDMS is −4.5 × 10^−4^/°C, and the relationship between its refractive index ($n_{PDMS}$) and temperature (*T*) [25] can be expressed as follows:

(4) $$n_{PDMS}\left(T\right)=-4.5\times 10^{-4}\Delta T+1.4176$$

When the transmission constant of the SPP mode and the fundamental mode satisfies the matching condition, the two modes are coupled to excite the SPR. However, the effective refractive index of the SPP mode is greatly affected by the change of refractive index of the liquid to be measured, and the effective refractive index of the core mode is weakly affected, which leads to the resonance wavelength shifts when the two modes meet the wave vector matching. Therefore, we can detect the change in the effective refractive index of the liquid by measuring the offset of the resonant wavelength. The sensitivity *S*(*λ*) is one of the important parameters to evaluate the performance of the PCF sensor, and its definition [26] is as follows:

(5) $$S\left(\lambda \right)=\frac{\Delta \lambda_{peak}}{\Delta n_{S}}\left(\frac{\mathrm{nm}}{\mathrm{RIU}}\right)$$

Among them, *∆λ_peak_* is the offset of the resonant wavelength, and *∆n_s_* is the change of the refractive index of the liquid to be measured.

However, for PCF sensors, when the sensitivity is too high, it may lead to an increase in internal loss, resulting in too high a full width at half maxima (FWMH) value of the resonant peak, resulting in a decrease in the resolution of the sensor. Therefore, we define the quality factor figure of merit (FOM) to measure the characteristics of the transmission curve, which is as follows:

(6) $$FOM=\frac{S\left(\lambda \right)}{FWHM}$$

Among them, sensor resolution *R* = *∆n_s_* × *∆λ_min_*/*∆λ_peak_*.

In this design, channel 1 and channel 2 were filled with different sensitive materials. When the external magnetic field or temperature changes, the refractive index of the sensitive materials in the two channels also changes; thus, the resonance coupling phenomenon between the SPP mode and the fiber core mode changes, resulting in the resonance wavelength shift; that is, integrated detection of magnetic field and temperature can be realized by measuring the resonant wavelength offset of channel 1 and channel 2, respectively. In the wavelength measurement method, the calculation formula of sensitivity is as follows:

(7) $$K_{ch}\left(T\right)=\Delta \lambda_{ch}/\Delta T$$

(8) $$K_{ch}\left(H\right)=\Delta \lambda_{ch}/\Delta H$$

Therefore, the relationship between resonant wavelength offset, temperature change, and magnetic field intensity change is expressed by a two-parameter sensitivity matrix as follows:

(9) $$\left(\begin{array}{c} \Delta \lambda_{1}\\ \Delta \lambda_{2} \end{array}\right)\;=\left(\begin{array}{cc} \frac{\Delta \lambda_{1T}}{\Delta T} & \frac{\Delta \lambda_{1H}}{\Delta H}\\ \frac{\Delta \lambda_{2T}}{\Delta T} & \frac{\Delta \lambda_{2H}}{\Delta H} \end{array}\right)\left(\begin{array}{c} \Delta T\\ \Delta B \end{array}\right)=\left(\begin{array}{cc} K_{ch1}\left(T\right) & K_{ch1}\left(H\right)\\ K_{ch2}\left(T\right) & K_{ch2}\left(H\right) \end{array}\right)\left(\begin{array}{c} \Delta T\\ \Delta H \end{array}\right)$$

Among them, *∆λ_i,j_*, *i* = 1,2, *j* = *T*, *H* denotes the shift of the resonant wavelength caused by the change of temperature or magnetic field intensity in two channels. *∆T* represents the change in external temperature, and *∆H* represents the change in the intensity of the external magnetic field, *K_chi_*(*j*), *i* = 1, 2, *j* = *T*, *H*, denotes the temperature sensitivity and magnetic field sensitivity of the two channels, respectively.

The sensitivity matrix in Equation (9) is inverted. That is, the sensing matrix for calculating the change of temperature and magnetic field can be obtained, and the expression is as follows:

(10) $$\left(\begin{array}{c} \Delta T\\ \Delta H \end{array}\right)={\left(\begin{array}{cc} K_{ch1}\left(T\right) & K_{ch1}\left(H\right)\\ K_{ch2}\left(T\right) & K_{ch2}\left(H\right) \end{array}\right)}^{-1}\;\left(\begin{array}{c} \Delta \lambda_{1}\\ \Delta \lambda_{2} \end{array}\right)$$

From Equation (10), we can calculate the change of temperature and magnetic field by measuring the resonant wavelength’s offset and realizing the simultaneous detection of temperature and magnetic field.

## 3. Results and Discussion

In this paper, using the full vector finite element method, the solution region was divided into finite elements. The process of mathematical approximation numerically simulated the physical structure designed in this paper. In the simulation, the free triangle meshing method was adopted. The frame was divided into 74,803 domain units, of which the minimum element mass was 0.4486. The numerical analysis of a surface plasmon resonance D-type PCF sensor integrated with temperature, magnetic field intensity, and refractive index was carried out under the boundary condition of a perfectly matched layer (PML). By changing the test environment and using wavelength modulation, the drift of the resonant wavelength was measured, and the sensor function of multi-parameter integrated detection was realized.

### 3.1. Sensor Structure Optimization

To obtain better sensor performance, then we optimized the structure of the sensor. First of all, under the condition of keeping other parameters unchanged, we used the transition boundary conditions to optimize the structure of photonic crystal fiber, and finally, used OriginPro software to process the data. Next, the sensor’s design was optimized, including the air hole spacing *Λ*, the side polishing depth *d_h_*, the air hole diameter *d*, and the diameter *d*_*ch*1_ and *d*_*ch*2_ of channel 1 and channel 2.

**Figure 2:**
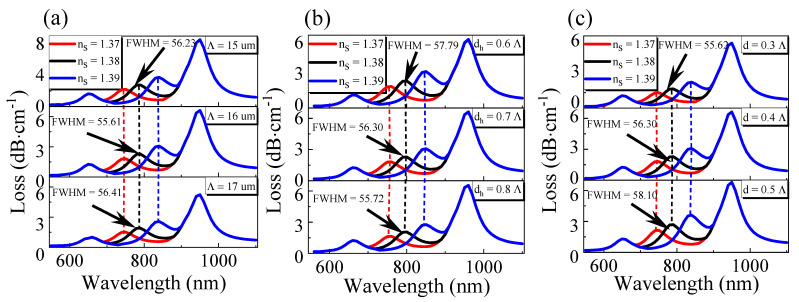
The variation of transmission loss with a wavelength and different refractive index of liquid to be measured: (**a**) with different air hole spacing *Λ*; (**b**) with various side’s depth *d_h_*; (**c**) with different air hole diameters *d*.

First of all, as can be seen from Figure 2a, the air hole spacing Λ changed from 15 µm to 17 µm and the loss decreased gradually. This is because the increase in the air hole spacing meant that the lattice period of the PCF became larger. The fiber had a larger core size, which made the core more restrained to light, more energy was constrained in the core, and the loss gradually became smaller. However, the change of air hole spacing did not affect the resonant wavelength of the sensor, but it caused the FWHM to increase at first and then decrease. Therefore, after a comprehensive analysis, we chose the air hole spacing *Λ* = 16 µm.

Then, the side depth d_h_ was optimized, and the simulation results are shown in Figure 2b. As shown in Figure 2b, when the side polishing depth d_h_ changed from 0.6 *Λ* to 0.8 *Λ*, the loss peak value decreased gradually, and the resonance wavelength was red-shifted. This is because, with the increase in *d_h_*, the distance between the core base mode and the SPW mode increases, resulting in a decrease in the coupling strength with the SPW mode and a reduction in the energy transferred from the core to the SPW mode, thus showing a peak decrease in the loss peak spectrum.** Although the change of *d_h_* will lead to the shift of resonance wavelength, the change curve of resonance wavelength is the same in different refractive index environments; therefore, the change of *d_h_* does not affect the sensitivity of the sensor, but it gradually reduces the FWHM of the sensor. Therefore, based on the above analysis, we finally chose the side depth *d_h_* = 0.7 Λ.

Then, we optimized the air hole diameter of the sensor, and the simulation results are shown in Figure 2c. The simulation results show that when the air holes diameter *d* changed from 0.3 Λ to 0.5 Λ, the loss of the sensor increased gradually. The reason is opposite to the reasons for the change in Figure 2a; the increase in the diameter of the air hole will lead to the smaller size of the fiber core, which will lead to the deterioration of the constraining ability of the fiber core to light. More energy will be transferred from the fiber core to SPW mode during resonance, and the loss will gradually increase.

Similarly, we can observe that the size of the air hole has no effect on the position of the resonant wavelength because the change of the air hole size has little impact on the core mode, so the phase-matching condition of SPR between the core mode and the SPW mode will not be affected. Therefore, we chose the diameter of the air hole as 0.5 Λ. Moreover, because the air hole spacing *Λ*, the side polishing depth *d_h_*, and the air hole diameter *d* do not affect the sensor’s sensitivity, the proposed sensor in this paper had a good fabrication tolerance.

**Figure 3:**
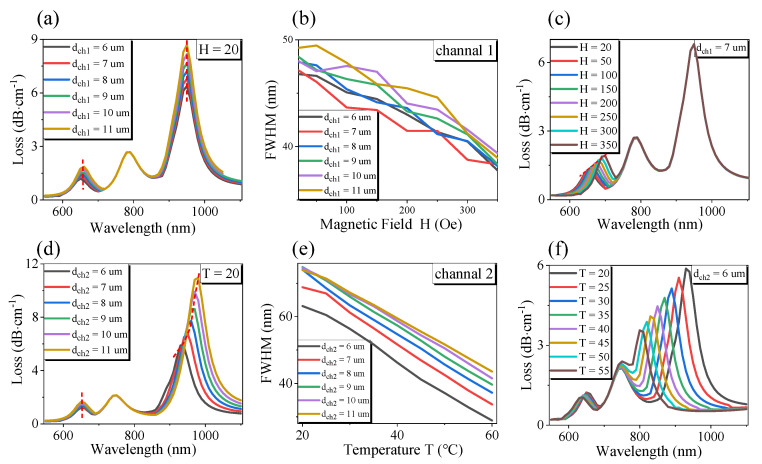
Structure optimization of plasmonic optical fiber sensor under the same refractive index of liquid to be measured: (**a**) The variation of transmission loss of channel 1 with wavelength; (**b**) The variation of FWHM with *H* under the same *T* and different channel 1 diameter; (**c**) The variation of transmission loss with wavelength at the same *T* and different *H* in *d*_*ch*1_ = 7 µm; (**e**) The variation of FWHM with *T* under the same *H* and different channel 2 diameters; (**f**) The variation of transmission loss with wavelength at the same *H* and different *T* in *d*_*ch*2_ = 6 µm.

Next, we continued to optimize the diameter of channel 1 and channel 2 of the sensor, and the simulation results are shown in Figure 3. From Figure 3a,d, we can see that when the diameter *d*_*ch*1_ and *d*_*ch*2_ increase, the loss of channel 1 and channel 2 increase gradually but have little effect on the RI. This is because when the diameter of channel 1 or channel 2 become larger, the defect hole diameter increases, making the distribution of the refractive index of the fiber more asymmetric and the birefringence of PCF stronger. It will also weaken the energy constraint ability of the fiber core in the *x*-axis direction, and more *x*-direction means more fundamental mode energy, which is coupled to channel 1 and channel 2, which increases the loss of channel 1 and channel 2. Simultaneously, the increase in channel 1 or channel 2 will improve the magnetic fluid content, and PDMS filled in channel 1 and channel 2 coupled more core energy to the channels on both sides. However, because the refractive index of MF is much smaller than that of the core, the energy coupled to channel 1 will be less than that of channel 2. At the same time, we can also find that the size changes of channel 1 and channel 2 do not affect the RI, which indicates that the size changes of the two channels have no effect on the base mode energy in the *y*-direction; when the diameter of the two channels increases, the metal film coated on the two channels will not only increase the contact area but also reduce the sensing distance from the fiber core diameter. However, the refractive index of MF is much smaller than that of the fiber core, so the shift of the resonant wavelength is not apparent, and the resonant wavelength is unchanged. However, the effect of channel 2 is more prominent. As shown in Figure 3d, the resonance wavelength will find a redshift, but under different diameters, the shifting trend of the resonance wavelength is the same; it has no effect on the sensor’s sensitivity. Additionally, from Figure 3b, it can be found that the increase in channel 1 size will make the FWHM decrease at first and then increase; Figure 3e shows that with the rise of channel 2 diameter, the FWHM will increase. When the magnetic field intensity changes, we can find from Figure 3c that the resonant wavelength of channel 1 changes, while RI and channel 2 are not affected. However, when the temperature changes, although channel 1 is mainly responsible for magnetic field detection because its filled magnetic fluid also has a temperature sensitivity coefficient, it will also have an impact on channel 1, as shown in Figure 3f. Similarly, temperature changes do not affect RI. This can reflect the multi-parameter integrated detection sensor we designed. There is no cross-influence among the parameters. Because *d*_*ch*1_ and *d*_*ch*2_ do not affect the sensor’s sensitivity, the sensor proposed in this paper has a good fabrication tolerance.

### 3.2. Graphene Layer Number

**Figure 4:**
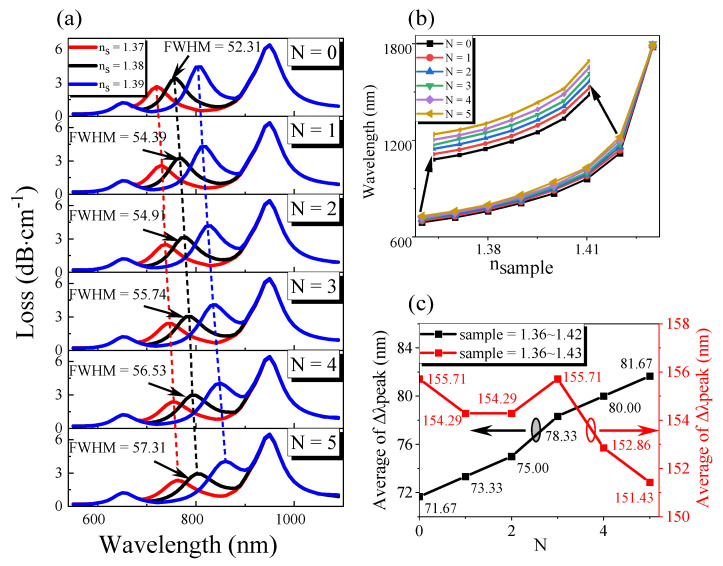
Optimization of graphene layer number of plasmonic optical fiber sensor under the condition of the same *T*, *H* and different refractive index of liquid to be measured: (**a**) The variation of transmission loss with wavelength under different graphene layers; (**b**) The change of resonance wavelength with the refractive index of the liquid to be measured under various layers of graphene; (**c**) The variation of the average resonant wavelength shift with the number of graphene layers under the different refractive index of the liquid to be measured.

Then, we used the transition boundary conditions to optimize the number of graphene layers while keeping other parameters unchanged. Finally, we used OriginPro software to process the data, and the results are shown in Figure 4. As shown in Figure 4a,b, in the same external environment, with the increase in graphene thickness, the resonance wavelength increases, the loss decreases, and the resonance curve widens, and FWHM increases. This is mainly because the effective refractive index of the metal interface increases with the increase in graphene thickness, so a higher resonance wavelength is needed to meet the resonance condition. At this time, the damping loss of graphene increases. Therefore, when SPR occurs, the core mode’s energy coupled to the SPP film is reduced, resulting in a reduced loss. From Figure 4c, we can see that when the refractive index of the external environment is in the range of 1.36 to 1.42, the average resonance wavelength shift increases with the increase in graphene thickness, so the average sensitivity of the sensor increases. When the refractive index of the external environment is in the range of 1.36 to 1.43, the average resonant wavelength shift fluctuates slightly with the rise of graphene thickness, reaches the maximum at *N* = 3, and then decreases gradually. This is because graphene is a kind of lossy medium with real and imaginary parts. When the number of layers of graphene (*N*) increases, the imaginary part of graphene also increases exponentially, which increases the carrier concentration and leads to the increase in the chemical potential (µ_c_) of graphene, and the corresponding resonance wavelength is red-shifted [27]. After a comprehensive analysis, we chose the layer number of graphene *N* = 3.

### 3.3. Gold Film Thickness

The thickness of the gold film has a significant influence on the PCF sensor’s sensing characteristics based on SPR. This is mainly because the energy of the evanescent wave excited by the fiber core attenuates exponentially during propagation, so the thickness of the gold film is smaller than the penetration depth of an evanescent wave *d_p_*. Among them, the formula of penetration depth *d_p_* [28] is as follows:

(11) $$d_{p}=\frac{\lambda }{2\pi {\left(n_{1}^{2}\sin^{2}\theta_{int}-n_{aq}^{2}\right)}^{1/2}}$$

Among them, *n*_1_ is the refractive index of the fiber core, *n_aq_* is the refractive index of the surrounding medium, and *θ_int_* is the incident angle. This paper is mainly designed in the visible and near-mid-infrared frequency band. As can be determined from Equation (11), the thickness of the metal film of this design is at least less than 60.5 nm. This design optimizes the thickness of a gold film from 25~60 nm, which corresponds with the propagation of an evanescent wave.

**Figure 5:**
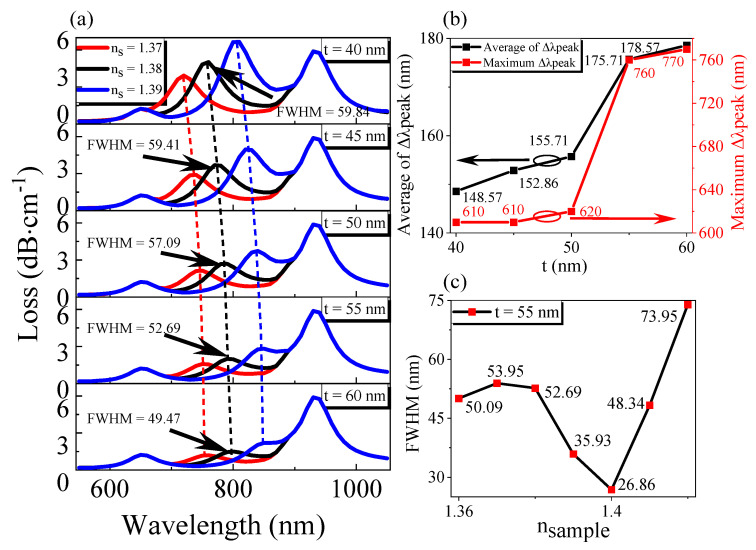
Optimization of metal film thickness *t* of plasmonic optical fiber sensor under the condition of the same *T* and *H*, and different refractive index of liquid to be measured: (**a**) The variation of transmission loss with wavelength; (**b**) The variation of resonance wavelength sensitivity of RI channel with gold film thickness *t*; (**c**) The FWHM of RI channel varies with the refractive index of the external environment for *t* = 55 nm.

We simulated the gold film thickness of the side polishing plane, channel 1, and channel 2. Firstly, the structure of the gold film thickness t coated on the side polishing plane was optimized, and the result is shown in Figure 5. As can be seen from Figure 5a, when the thickness t of the gold film becomes more extensive, the loss of the sensor decreases gradually, the resonance wavelength is red-shifted, and the FWHM decreases slowly. This is mainly because the thicker the gold film is, the more the energy of evanescent wave attenuates and less energy is coupled with the SPW mode, so the loss becomes smaller. When the thickness of the gold film increases, the effective refractive index of SPW increases, but the core remains unchanged, which the phase matching point of SPW mode and the core fundamental mode redshifts when SPR occurs, that is, the resonance wavelength redshifts. In this case, FWHM decreases with the increase in the gold film thickness because when the gold film is relatively thin, the evanescent field’s energy focused on the film is very weak, and the excitation effect of SPR is not good, thus making the FWHM larger. Besides, it can be found that when the thickness of the gold film changes or when the refractive index changes, channel 1 and channel 2 will not change, indicating that the RI channel will not have cross-effects with the other two channels.

When the refractive index *n_s_* changes successively from 1.36 to 1.43 in turn by step length 0.01, and the thickness of the gold film varies from 40 nm to 60 nm in turn by step length 5 nm, from Figure 5b, we know that the average resonant wavelength shift and maximum resonant wavelength shift gradually increase. Based on the above analysis, the gold film thickness *t* = 55 nm was selected in this paper. In this case, the average resonant wavelength offset is 175.71 nm, and the maximum resonant wavelength offset is 760 nm. According to Formula (7), the average sensitivity of the sensor designed in this paper is 17,571 nm/RIU. Maximum sensitivity can reach 76,000 nm/RIU. When the minimum resolution of the spectrometer is *Δλ_min_* = 0.1 nm, the average minimum spectral resolution is 5.69 × 10^−6^ (RIU). From Figure 5c, the minimum FWHM of RI can reach 26.86 nm at *t* = 55 nm. According to Formula (6), the FOM corresponding to the average sensitivity and the maximum sensitivity are 654.17 RIU^−1^ and 2829.49 RIU^−1^.

**Figure 6:**
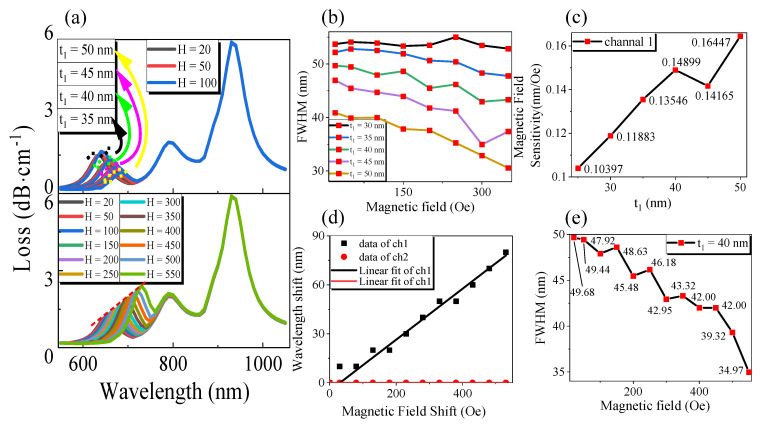
Optimization of metal film thickness *t*_1_ structure of plasmonic optical fiber sensor under the same refractive index of *T* and liquid to be measured: (**a**) The variation of transmission loss with wavelength; (**b**) The FWHM of channel 1 varies with *H* under different thicknesses of *t*_1_; (**c**) The variation of magnetic field intensity sensitivity with *t*_1_; (**d**) The variation of resonant wavelength shift of channel 1 and channel 2 with magnetic field intensity and its linear fitting results; (**e**) The FWHM of channel 1 varies with the intensity of the magnetic field for *t*_1_ = 40 nm.

Next, the structure of the gold film thickness *t*_1_ of channel 1 was optimized, and the result is shown in Figure 6. It can be seen from Figure 6a,b, when the thickness of the gold film *t*_1_ becomes larger, channel 1 affects the *x*-direction fundamental mode of the fiber core, so that the loss of the sensor decreases gradually, the resonance wavelength is redshifted, and the FWHM decreases slowly. However, the increase in t_1_ does not affect the symmetrical channel 2 in the *x*-direction. This is because the change of the thickness of the gold film in the two channels has little effect on the energy constraint ability of the core in the *x*-axis direction. With the increase in magnetic field intensity, the magnetic particles of MF gather, and the effective refractive index increases, which leads to the change of phase matching point during resonance coupling, the red shift of resonance wavelength, the better effect of SPR excitation, and the decrease in FWHM. At this point, from Figure 6a,b, we can see that channel 2 and the RI channel have not changed, indicating that channel 1 will not have a cross effect with the RI channel and channel 2. Under different magnetic field ranges, when the gold film thickness t_1_ changes sequentially from 25 nm to 50 nm in turn by step length 5 nm, the magnetic field sensitivity tends to increase by observing the Figure 6c. Because when the gold film is too thin, the SPR excitation effect is not good, the FWHM is too high, the gold film is too thick, and the loss peak is too low to be observed. With comprehensive consideration, the designers chose channel 1 gold film thickness *t*_1_ = 40 nm.

When the magnetic field intensity varies at 20~550 Oe, Figure 6d shows that the resonant wavelength shift increases with the magnetic field intensity offset. At this time, through the linear fitting of OriginPro software, we can obtain *Δλ_1H_* = 0.15631*ΔH* − 4.89956, the sensitivity of magnetic field intensity was 156.31 pm/Oe, and the degree of linearity was 0.99027; the wavelength resolution of the sensor was assumed to be *Δλmin* = 0.1 nm, and the magnetic field resolution was 0.64 Oe [29]. Channel 2 was not affected by the change of magnetic field intensity. As shown in Figure 6e, when *t*_1_ = 40 nm, the FWHM of channel 1 decreased with the increase in magnetic field intensity, and the minimum can reach 34.97 nm. In this case, according to Equation (6), FOM = 4.47 × 10^−3^ Oe^−1^.

**Figure 7:**
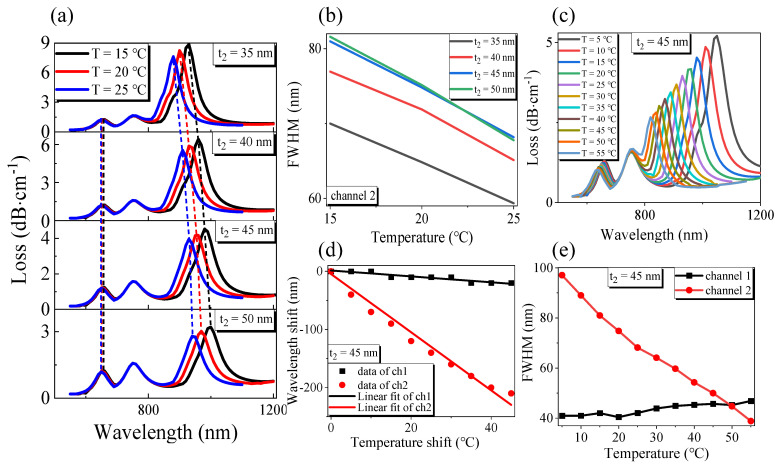
Optimization of metal film thickness *t*_2_ structure of plasmonic optical fiber sensor under the same refractive index of *H* and liquid to be measured: (**a**) The variation of transmission loss with wavelength; (**b**) The FWHM of channel 1 and channel 2 varies with *T* under the different thickness of *t*_2_; (**c**) The variation of transmission loss with wavelength at different T for *t*_2_ = 45 nm; (**d**) The variation of resonant wavelength shift of channel 1 and channel 2 with *T* and its linear fitting results; (**e**) The FWHM of channel 1 and channel 2 varies with the intensity of T for *t*_2_ = 45 nm.

Finally, the structure of the gold film thickness *t*_2_ of channel 2 was optimized, and the result is shown in Figure 7. As shown in Figure 7a,b, when *t*_2_ increases, channel 2 influences the primary mode in the *x*-direction of the fiber core, making the loss of channel 2 gradually decrease, the resonance wavelength red-shift, and a gradual increase in FWHM. Simultaneously, the rise of *t*_2_ does not affect the symmetrical channel 1 in the *x*-direction. Figure 7a,b shows that channel 2 and RI channels have not changed, indicating that channel 1 will not have cross effects with RI channels and channel 2. However, the thickness of the gold film *t*_2_ will affect the distance between the RI channel’s resonant wavelengths and channel 2. When the space is too close, it will lead to the superposition of the two resonant wavelengths. With comprehensive consideration, the designers chose the channel 1 gold film thickness *t*_2_ = 45 nm.

The temperature *T* is changed sequentially from 5 °C to 55 °C, with a step size of 5 °C for *t*_2_ = 45 nm. By observing Figure 7c, it can be found that both channel 1 and channel 2 have a blue shift in resonance wavelength and a gradual decrease in the loss with the increase in temperature. This is because both MF and PDMS of channel 1 and channel 2 have negative temperature coefficients. Figure 7d shows that the resonant wavelength offset of the two channels decreases with the increase in the temperature shift; after the linear fitting of OriginPro software, the linear fitting expression of channel 1 could be obtained as *Δλ_1T_* = −0.50909*ΔT* + 1.45455, the temperature sensitivity was −509.09 pm/°C, the degree of linearity was 0.95455, the linear fitting expression of channel 2 was *Δλ_2T_* = −5.00131*ΔT* − 4.73636, the temperature sensitivity was 5001.31 pm/°C, and the degree of linearity was 0.99275. The wavelength resolution of the sensor was assumed to be *Δλmin* = 0.1 nm, and the temperature resolutions of channel 1 and channel 2 were 0.196 °C and 0.02 °C, respectively. From Figure 7e, when *t*_2_ = 45 nm, the FWHM of channel 1 increased with the increase in temperature, the minimum FWHM was 40.43 nm, and the FWHM of channel 2 decreased with the rise of temperature, and the minimum could reach 38.84 nm. According to Equation (6), the FOM of channel 1 and channel 2 are −1.26 × 10^−2^ °C^−1^ and −0.129 °C^−1^, respectively.

Through the above analysis, we obtain the temperature sensitivity of the sensor channel 1 and channel 2 as −509.09 pm/°C and −5001.31 pm/°C, and magnetic field intensity sensitivity as 156.31 pm/Oe and 0 pm/Oe, respectively. According to Equation (9) and Equation (10), the sensing matrix for detecting the change of external temperature and magnetic field intensity can be obtained:

(12) $$\left(\begin{array}{c} \Delta T\\ \Delta H \end{array}\right)={\left(\begin{array}{cc} -0.50909 & 0.15631\\ -5.00131 & 0 \end{array}\right)}^{-1}\left(\begin{array}{c} \Delta \lambda_{1}\\ \Delta \lambda_{2} \end{array}\right)=\left(\begin{array}{cc} 0 & -0.19995\\ 6.39754 & -0.65121 \end{array}\right)\left(\begin{array}{c} \Delta \lambda_{1}\\ \Delta \lambda_{2} \end{array}\right)$$

To summarize, the plasmonic optical fiber sensor proposed in this paper, which integrates temperature, magnetic field intensity, and refractive index multi-parameter detection, can not only detect these three parameters at the same time, but also has high refractive index sensitivity, temperature sensitivity, and magnetic field sensitivity.

## 4. Conclusions

In summary, a surface plasmon resonance D-type photonic crystal fiber (PCF) sensor integrated with multi-parameter detection of temperature, magnetic field intensity, and refractive index has been proposed. The theoretical model was simulated and analyzed by the finite element method. The D-type photonic crystal fiber SPR sensor for multi-parameter integrated detection was realized using the magneto-optic effect of magnetic fluid and the temperature-sensitive development of SPR and PDMS. The advantages of this design are: it can effectively avoid the cross-sensitivity among the three parameters, realize the integrated detection of multi-parameters, overcome the limitation of single-parameter measurement of traditional sensors, and provide an idea for the field of multi-function sensor detection. The graphene layer was introduced to improve the RI channel’s sensitivity so that the average sensitivity of the designed RI was 17,571 nm/RIU, and the maximum sensitivity was 76,000 nm/RIU, which was higher than that of some other published sensors [30,31,32]. This design has the advantages of a simple structure and principle, and high magnetic field sensitivity and temperature sensitivity in visible and near-mid-infrared bands. This design also has good production tolerance. Therefore, the sensor designed in this paper is a promising tool in the field of multi-sensor detection and biosensor.

## Data Availability

Data sharing not applicable.
